# Editorial: Mechanopathology: unraveling the mechanical forces driving disease mechanisms

**DOI:** 10.3389/fcell.2026.1789070

**Published:** 2026-02-12

**Authors:** Cristina Bertocchi, Emanuele Rizzuto, Esther W. Gomez, Barbara Peruzzi

**Affiliations:** 1 Faculty of Biological Sciences, Pontificia Universidad Católica de Chile, Santiago, Chile; 2 Graduate School of Engineering Science, Osaka University, Osaka, Japan; 3 Department of Mechanical and Aerospace Engineering, Sapienza University of Rome, Rome, Italy; 4 Department of Chemical Engineering, The Pennsylvania State University, University Park, PA, United States; 5 Department of Life Science and Public Health, Section of Histology and Embryology, Catholic University of Sacred Heart, Rome, Italy; 6 Oncology and Experimental Neuroscience Unit, IRCCS Fondazione Policlinico “Agostino Gemelli”, Rome, Italy

**Keywords:** actin cytoskeleton, disease, forces, mechanopathology, mechanotransduction, tumor microenvironment

Mechanical forces are essential regulators of cellular and tissue function, actively shaping behavior, gene expression, and tissue organization. When altered, these physical cues, such as tissue stiffness, shear stress, and mechanical strain, can drive pathological scenarios. This dynamic interplay defines mechanopathology, where abnormal mechanical environments and defective mechanosensing, alongside biochemical and genetic factors, directly contribute to disease initiation and progression.

This Research Topic integrates five multidisciplinary contributions that illustrate how mechanical forces influence molecular and cellular mechanisms across pathological contexts, including inflammation, vascular and lymphatic biology, cancer, and musculoskeletal degeneration, while highlighting their potential to inspire innovative diagnostic and therapeutic strategies.

A unifying theme emerges from these studies: mechanical cues are not merely modulators of disease but active drivers that integrate with biochemical signaling to shape pathological outcomes. At the cellular scale, the actin cytoskeleton, dynamically regulated by polymerization complexes such as the actin-related protein 2/3 (Arp2/3) complex, represents a primary interface between mechanical forces and intracellular signaling. In their comprehensive review, Xing et al. link Arp2/3-mediated-actin polymerization dynamics to immune cell migration, phagocytosis, and cytokine production. Their findings reveal that cytoskeletal remodeling, driven by Arp2/3, enables immune cells to reorganize actin into branched networks, critical for forming lamellipodia and filopodia that interact with the extracellular matrix (ECM). This interaction is not passive: the ECM’s physical properties, such as stiffness and topography, feedback to modulate Arp2/3 activity, creating a bidirectional mechanotransduction loop that fine-tunes immune responses. For example, in stiffened ECM environments, such as those found in fibrotic tissues or tumors, Arp2/3 activation is enhanced, leading to increased immune cell infiltration and pro-inflammatory cytokine release, which further remodels the ECM.

Intriguingly, this principle extends into vascular and lymphatic biology, where cells are constantly exposed to dynamic physical forces. Xu et al. provide experimental evidence of how ECM stiffness modulates the proliferation and migratory behaviour of lymphatic endothelial cells through the mechanosensitive protein FAT (FAT Atypical Cadherin) 1. Their findings reveal that FAT1 acts as a pivotal mechanosensor, translating ECM stiffness into intracellular signals that regulate lymphatic endothelial cell behaviour and tissue homeostasis. This mechanotransduction pathway underscores how altered tissue mechanics, whether due to fibrosis, chronic inflammation, or tumor growth, actively drive pathological processes, reinforcing the notion that ECM physical properties are not merely consequences of disease, but central regulators of cellular behaviour and tissue homeostasis.

This is best embodied in cancer, where abnormal tissue mechanics (i.e., ECM stiffness, solid stress, and interstitial pressure) are a hallmark of the tumor microenvironment (TME). In their review, Angeli et al. explore how, in solid tumors such as breast carcinoma and melanoma, physical characteristics of the TME, orchestrate tumor growth, invasion, immune cell infiltration, and treatment resistance. These interconnected physical forces drive tumor progression by activating mechanosensitive pathways, including Yes-associated protein (YAP)/transcriptional coactivator with a PDZ-binding domain (TAZ) signaling and integrin-mediated cytoskeletal reorganization. Within this stiffened, pressurized TME vascular and lymphatic compression exacerbates hypoxia and immune evasion, while also triggering oncogenic programs that enhance cell survival, migration, and resistance to therapy. YAP/TAZ, acting as central mechanotransducers, amplify these effects by upregulating targets like cysteine-rich angiogenic inducer 61 (CYR61) and connective tissue growth factor (CTGF) and modulating immune checkpoint expression (e.g., Programmed Death Ligand 1, PD-L1), thus linking ECM mechanics to both tumor cell autonomy and immune suppression. This mechanistic framework underscores the translational potential of targeting mechanical cues, through YAP/TAZ inhibition, anti-fibrotic agents, or Focal Adhesion Kinase (FAK)/Piezo1 blockade, to disrupt the pro-tumorigenic feedback loop between the ECM and cancer cells.

Expanding on these observations, Zhang et al. offer a comprehensive review of mechanosensitive ion channels (such as Piezo and transient receptor potential (TRP) family members) focusing on their role in osteoarthritis pathogenesis. The authors summarize current knowledge on these channels emphasizing their ability to convert mechanical stimuli (e.g., compressive stress, shear forces) into intracellular signals that drive inflammation, ECM degradation, and pain sensitization. Piezo1/2 and TRPV4, upregulated in osteoarthritic chondrocytes, trigger calcium influx, matrix metalloproteinases (MMP)/tissue inhibitors of metalloproteinases (TIMP) imbalance, and chondrocyte senescence, thereby accelerating cartilage breakdown. Chondrocyte-specific knockout of Piezo1 and Piezo2 in murine models attenuates post-traumatic osteoarthritis progression, highlighting these channels as therapeutic targets for both structural preservation and symptom relief.

In the context of post-traumatic osteoarthritis (PTOA), Miao et al. investigate the mechanosensitive protein Anthrax toxin receptor (ANTXR)1 revealing its complementary role in maintaining cartilage homeostasis. Their work demonstrates that ANTXR1 interacts with the Wnt co-receptor Low-density lipoprotein receptor-related protein 6 (LRP6) to maintain cartilage homeostasis under mechanical stress, and that its deficiency aggravates cartilage degeneration following injury. Together, these studies frame osteoarthritis as a disease of dysregulated mechanotransduction, where Piezo1/2/TRPV4 drive degradation and inflammation, and ANTXR1 supports cartilage integrity, offering dual therapeutic avenues: inhibiting ion channels to block catabolic pathways, and restoring ANTXR1 function to promote anabolic repair.

Taken together, the articles in this Research Topic converge on several unifying principles. First, mechanical forces are integrated within cellular signaling networks, regulating processes from cytoskeletal dynamics to ion channel activity and gene expression. Second, pathological alterations in tissue mechanics actively drive disease progression, as demonstrated across inflammation, vascular biology, osteoarthritis, and cancer. Third, these mechanotransduction pathways offer targets for therapeutic intervention, where mechanical and biochemical signals intersect. In summary, this Research Topic positions mechanopathology as a unifying framework for understanding disease, reinforcing the importance of mechanics alongside genetic and biochemical factors.

